# ASSOCIATION OF DIABETES WITH OLFACTION IN OLDER ADULTS: THE ATHEROSCLEROSIS RISK IN COMMUNITIES (ARIC) STUDY

**DOI:** 10.1093/geroni/igae098.2398

**Published:** 2024-12-31

**Authors:** Hanna Pleasants, Honglei Chen, Tom Mosley, Keran Chamberlin, Yaqun Yuan, Srishti Shrestha, Jayant Pinto, Anna Kucharska-Newton

**Affiliations:** University of North Carolina, Chapel Hill, North Carolina, United States; Michigan State University, Michigan State University, Michigan, United States; University of Mississippi Medical Center, Jackson, Mississippi, United States; Michigan State University, United States; Michigan State University East Lansing, East Lansing, Michigan, United States; University of Mississippi Medical Center, Jackson, Mississippi, United States; University of Chicago, Chicago, Illinois, United States; University of North Carolina at Chapel Hill, Chapel Hill, North Carolina, United States

## Abstract

Although extant research suggests a cross-sectional association between prevalent diabetes and poor olfaction, the long-term association of diabetes with olfactory impairment remains unclear. We examined the association of diabetes in midlife and in older adulthood, with olfaction ascertained in older adulthood among 6,122 participants of the ARIC cohort who attended ARIC Visit 5 (2011-2013; 58.6% female, 22.7% Black race). Diabetes status and hemoglobin A1c (HbA1c) levels were ascertained at midlife (Visit 2; 1990-1993; mean age 55.0 (SD 5.2)) and in older adulthood (Visit 5; mean age 75.8 (SD 5.2) years). Odor identification was measured at Visit 5 using the 12-item Sniffn’ Sticks and categorized as poor (0-8) versus moderate or good (9-12). Binary logistic regression (exposure: diabetes status) and linear regression (exposure: HbA1C level) were used to model hypothesized associations. Diabetes prevalence at midlife (Visit 2) was associated with poor as compared to good olfaction in older adulthood (median follow-up time 21 years; adjusted odds ratio (AOR) (95% CI) = 1.25 (1.02, 1.53)). Similarly, greater HbA1c in midlife was associated with a lower olfaction score in older adulthood (beta coefficient: -0.101 (SE ± 0.04) per each 1 percent greater HbA1c level). Observed associations were attenuated when diabetes and HbA1c, in association with olfaction, were ascertained in older adulthood (all at Visit 5) (AOR 1.14 (95% CI: 1.00, 1.29); beta coefficient: -0.06 (SE ± 0.04), respectively). These findings support the association between midlife diabetes and impairment in olfactory function in older age.

